# Fluoroacetate in plants - a review of its distribution, toxicity to livestock and microbial detoxification

**DOI:** 10.1186/s40104-017-0180-6

**Published:** 2017-06-01

**Authors:** Lex Ee Xiang Leong, Shahjalal Khan, Carl K. Davis, Stuart E. Denman, Chris S. McSweeney

**Affiliations:** 1 School of Chemistry and Molecular Bioscience, University of Queensland, St Lucia, 4072 QLD Australia; 2 School of Agriculture and Food Sciences, University of Queensland, St Lucia, 4072 QLD Australia; 3 CSIRO Agriculture and Food, Queensland Bioscience Precinct, St Lucia, 4072 QLD Australia

**Keywords:** Aerobic, Anaerobic, Degradation, Dehalogenase, Fluoroacetate, 1080, Synergistetes, TCA, Toxicity

## Abstract

Fluoroacetate producing plants grow worldwide and it is believed they produce this toxic compound as a defence mechanism against grazing by herbivores. Ingestion by livestock often results in fatal poisonings, which causes significant economic problems to commercial farmers in many countries such as Australia, Brazil and South Africa. Several approaches have been adopted to protect livestock from the toxicity with limited success including fencing, toxic plant eradication and agents that bind the toxin. Genetically modified bacteria capable of degrading fluoroacetate have been able to protect ruminants from fluoroacetate toxicity under experimental conditions but concerns over the release of these microbes into the environment have prevented the application of this technology. Recently, a native bacterium from an Australian bovine rumen was isolated which can degrade fluoroacetate. This bacterium, strain MFA1, which belongs to the Synergistetes phylum degrades fluoroacetate to fluoride ions and acetate. The discovery and isolation of this bacterium provides a new opportunity to detoxify fluoroacetate in the rumen. This review focuses on fluoroacetate toxicity in ruminant livestock, the mechanism of fluoroacetate toxicity, tolerance of some animals to fluoroaceate, previous attempts to mitigate toxicity, aerobic and anaerobic microbial degradation of fluoroacetate, and future directions to overcome fluoroacetate toxicity.

## Background

Sodium monofluoroacetate (referred to as fluoroacetate hereafter), has the chemical formula FCH_2_COO^−^Na^+^, and is a highly toxic compound primarily used as a pesticide known commercially as Compound 1080. Despite having a strong carbon-fluorine bond (one of the strongest bonds in nature), fluoroacetate appears to be rather labile in the environment being readily degraded by different microorganisms [1] or anabolised by higher organisms. This is in contrast to polyfluorinated compounds (such as Teflon) which are very recalcitrant and can persist in the environment for many years [2]. It is well suited as a pesticide because it is virtually tasteless and odourless, which enables it to be easily disguised within bait material targeted towards a specific pest species [3]. However, due to its non-specific poisoning of other animals and accidental human ingestion, this pesticide is currently used under strict control by governments around the world.

Fluoroacetate was first synthesised in the laboratory in 1896 but it was only first isolated from “gifblaar” (a South African plant) by Marais in 1943 [4]. These plants were believed to naturally produce this toxic compound as a defence mechanism against grazing by herbivores. Ingestion by livestock often results in fatal poisonings, which causes significant economic problems to commercial farmers in many countries such as Australia, Brazil and South Africa [5–8]. In Brazil, 60% of the cattle losses are due to fluoroacetate poisoning from grazing fluoroacetate-producing plants [9]. Fluoroacetate toxicity costs the Australian livestock industry around 45 million dollars (AUD) annually due to the increased death rates and associated productivity impacts [10]. In this paper, we will focus on the natural fluoroacetate found in plants impacting ruminant livestock industries, mechanism of its toxicity, previous attempts to mitigate toxicity, aerobic and anaerobic microbial degradation of fluoroacetate, tolerance of some animals to fluoroaceate, and future directions to overcome fluoroacetate toxicity.

## Fluoroacetate in the environment

Fluoroacetate containing plants grow worldwide and cause sudden death in livestock. The southern continents of Africa, Australia and South America are the common locations of these plants. All of the plants containing fluoroacetate belong to the families Fabaceae, Rubiaceae, Bignoniaceae, Malpighiaceae and Dichapetalaceae [11].

Fluoroacetate is found in these tropical and subtropical plants generally at low concentrations although some are able to accumulate fluoroacetate in high concentrations [12]. These plants grow on a variety of soil types, including acidic, heavier soils or sandy loams but rarely in deep sandy soil [7]. In Africa, most fluoroacetate-accumulating plants belong to the genus *Dichapetalum*. The seeds of *D. braunii* can contain levels of fluoroacetate up to 8000 mg/kg, which is the highest ever recorded [13]. Fluoroacetate is also present in plants from South America, particularly *Palicourea marcgravii*, which can contain levels up to 500 mg/kg [14]. Other South American plants that are known to contain fluoroacetate are from the *Amorimia* genus, which has lower concentration of fluoroacetate than *P. marcgravii* [15]. Although plants from South America may not contain high concentration of fluoroacetate, they are still responsible for many livestock deaths due to the high toxicity of fluoroacetate.

**Fig. 1:**
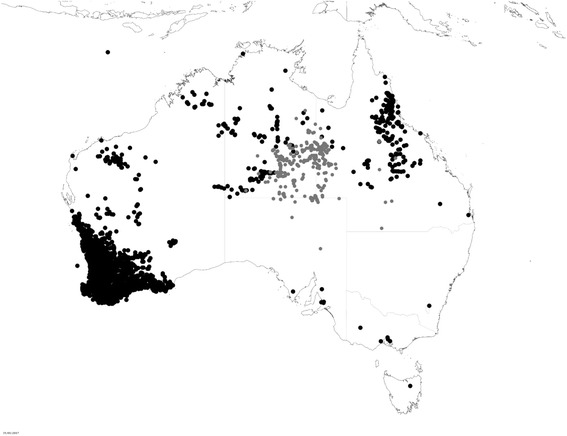
Distribution of fluoroacetate bearings plants in Australia. Black dots Gastrlobium spp., grey dots Acaia georginae, generated from the Atlas of Living Australia 15/05/2017 (http://www.ala.org.au/)

In Australia, about 40 species of plants can generate fluoroacetate and most of them belong to the genus *Gastrolobium* [16]. Later these plants were classified as three genera *Gastrolobium, Oxylobium* and *Acacia*. After reclassification, many of the “nontoxic” *Gastrolobium* spp. haven been transferred to the genus *Nemcia* and the “toxic” *Oxylobium* spp. have all been placed in *Gastrolobium* [17, 18]. These fluoroacetate-containing plants are widely distributed in Australia (Fig. 1). Heart-leaf bush, *Gastrolobium grandiforum*, can contain as much as 2600 mg/kg fluoroacetate, while the 50% lethal dose (LD_50_) of fluoroacetate is only 0.4 mg/kg of cattle body weight [12]. Although it contains less fluoroacetate than some other species, they are responsible for most of the livestock deaths in Australia because of their high abundance in cattle-producing regions [19].

In South America, especially in Brazil, around 500,000 cattle die every year by poisonous plants which cause sudden death [20]. *Palicourea marcgravii* and *Amorimia rigida* are the two most common toxic plants in Brazil [21]. Fluroacetate was found to be the principle toxin in these two plants [22]. In South Africa, *Dichapetalum cymosum* is the third most important poisonous plant causing livestock deaths particularly during spring and episodes of drought [23]. The biosynthesis pathway of fluoroacetate by these plants is still largely unknown. This is the result of the inability to produce stable fluoroacetate-degrading plant cell lines [24, 25]. Although a cell-free extract of *Dicepatalum cymosum* is able to convert fluoropyruvate to fluoroacetate, researchers could not identify the mechanism and enzymes required [26]. Analysis of soils in which some fluoroacetate-accumulating plants are found show that biosynthesis of fluoroacetate occurs even when total soil inorganic fluoride is very low [14]. Fluoroacetate biosynthesis seems to be relatively widespread, however some plants clearly have evolved to accumulate high concentrations, giving them a selective advantage from predation by animals.

This review will focus mainly on toxicity of fluoroacetate but some plants also contain fluorocitrate, fluoroacetone and fluorofatty acid compounds. Fluorinated natural products, for example, the seeds of *Dichapetalum toxicarium*, an indigenous shrub of West Africa, cause death of animals after ingestion and the symptoms are similar to fluoroacetate poisoning [27]. The seeds of *D. toxicarium* contain up to 1800 μg/g  organic fluorine and the main fluorinated component was ω-fluorooleic acid (C18:1 F) [28]. Additional fluorofatty acids including o ~ −fluoro-palmitoleic, -stearic, -linoleic, -arachidic and -eicosenoic acids and 18-fluoro-9,10-epoxystearic acid have since been identified [29].

**Fig. 2:**
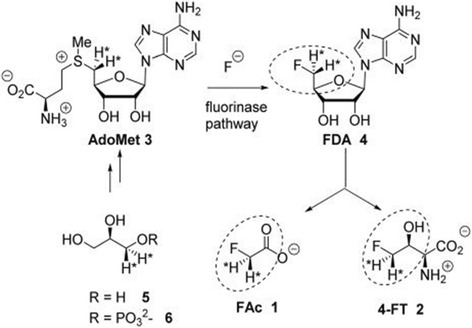
Production of 5´-fluorodeoxyyadenosine (FDA) from S-adenosyl-L-l-methionine (Adomet) by Fluorinase reaction (3–4). Formation of Fluoroaceate (FAc) and 4-fluorothreonine (4-FT) from (4 to 1–2). Incorporation of Isotope labelled Glycerol (5 and 8 to 3)

Some bacteria have been identified that can produce fluoroacetate in the environment. For example the soil bacterium *S. cattleya,* possess fluorinase (fluorination enzyme) which catalyses a nucleophilic substitution reaction between fluoride ion and S-adenosyl-L-methionine to produce 5′-fluorodeoxyadenosine (FDA). FDA is then processed to fluoroacetate and 4 -fluorothreonine (4-FT). By incorporating isotopically labelled glycerol it has been determined that the C5′ fluoromethyl and C4′ carbon of FDA are converted to fluoroacetate and C3 and C4 of 4-FT. It has also been established that both hydrogens of the fluoromethyl group of FDA are reserved in the conversion to the fluoromethyl groups of fluoroacetate and 4-FT [30] (Fig. 2).

## Fluoroacetate toxicity mechanism

**Fig. 3:**
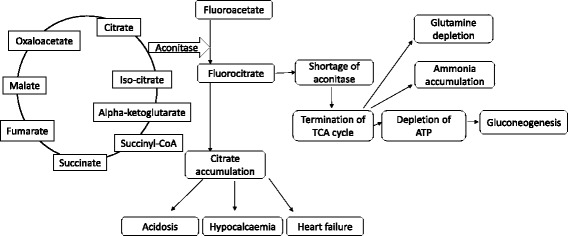
Mechanisms of fluoroacetate toxicity

The tricarboxylic acid (TCA) cycle is central to cellular energy production in the mitochondria of higher organisms and fluoroacetate interrupts the TCA cycle. Fluoroacetate poisoning has been well-documented in animals since its application as a pesticide. Following oral administration and absorption through the gut, fluoroacetate is converted to fluorocitrate by citrate synthase (EC 4.1.3.7) [31] which strongly binds to the aconitase enzyme (EC 4.2.1.3), that converts citrate to succinate in the citric acid cycle [31]. This results in the termination of cellular respiration due to a shortage of aconitase [32, 33], and an increase in concentration of citrate in body tissues including the brain [32]. The build-up of citrate concentration in tissues and blood also causes various metabolic disturbances, such as acidosis which interferes with glucose metabolism through inhibition of phosphofructokinase, and citric acid also binds to serum calcium resulting in hypocalcaemia and heart failure [32, 34–37] (Fig. 3).

Despite a common mechanism of poisoning in all vertebrates, there are differences in the signs and symptoms of fluoroacetate toxicity. In general, carnivores (dogs) show primarily central nervous system (CNS) signs including convulsions and running movements with death due to respiratory failure. Herbivores (rabbit, goat, sheep, cattle, horse) show mostly cardiac effects with ventricular fibrillation and little or no CNS signs. The clinical symptoms of omnivores similarly consist of both cardiac and respiratory failure and central nervous system depression [38].

In the pig (omnivores), the clinical symptoms consist of ventricular fibrillation, tremors, violent myotonic convulsions, and respiratory depression [39]. Moreover, the onset of these symptoms can vary between animals of the same species [3]. The symptoms of fluoroacetate poisoning in cattle consist of urinary incontinence, loss of balance, muscle spasms, and in-place running lasting 3 to 20 min or convulsion followed by death of the animal [40]. In Robison’s [40] report, symptoms were undetected for up to 29 h following ingestion of fluoroacetate and occurred just before death, hence the term “sudden death” described by some researchers [5]. The clinical symptoms of fluoroacetate poisoning in sheep are relatively similar to cattle, including abnormal posturing, urinary incontinence, muscle spasms and convulsions. They are also known to have severe respiratory distress and extremely rapid heart rate [39, 41].

Diagnosis is generally made on the basis of verified exposure, clinical signs, necropsy findings and chemical analysis. Samples for analysis are, vomitus, liver, stomach or rumen contents and kidney. Increased citric acid levels in kidney and serum is an indicator of fluoroacetate poisoning when correlated with clinical history. Differential diagnosis can be made amongst compounds such as strychnine, chlorinated hydrocarbons, plant alkaloids and lead. A number of other non-specific biochemical changes are suggestive including hyperglycaemia, hypocalcaemia, hypokalaemia and metabolic acidosis [10].

## Fluoroacetate tolerance

Many species of animal possess an innate tolerance to fluoroacetate even when there is no evidence of evolutionary exposure. Dogs and other carnivores and rodents and many wildlife species are highly susceptible. Mammalian herbivores have intermediate sensitivity. Reptiles and amphibians are the most tolerant within the animal kingdom. Fish are generally more resistant. This tolerance is likely due to the reduced metabolic rate of these animals. It has been demonstrated that a lower metabolic rate results in less fluoroacetate being converted to fluorocitrate thus allowing more time for excretion and detoxification [42]. The skink (*Tiliqua rugosa*) has a metabolic rate about 10 fold less than a rat of similar size, but has approximately 100 fold greater tolerance to fluoroacetate [43]. Mammals with lower metabolic rate such as the bandicoot also possess a greater tolerance to fluoroacetate [44].

Interestingly, some Australian animals that live in areas where there are fluoroacetate accumulating plants have acquired a remarkable tolerance to fluoroacetate [45, 46]. The degree of tolerance is most apparent in herbivores, especially seed eating birds, which are most likely to have more direct exposure to the toxin compared to carnivorous animals [47]. Other factors which influence the degree of tolerance within a species or population may include the length of time exposed to toxic vegetation, the broadness of both diet and habitat, the size of the resident habitat and the degree of mobility. The emu, which is Australia’s oldest seed eating bird, can be up to 150-times more tolerant than the same species of emu outside of areas with fluoroacetate-accumulating plants [48]. This phenomenon has also been observed in other animals such as the possum [42]. Tolerance to fluoroacetate is also demonstrated in insects. Some insects not only utilise the vegetation in their diet, but some actually store the toxin, probably in vacuoles, and use it as defence against predation [49].

The biochemical nature of acquired tolerance to fluoroacetate in animals is not fully understood. It is proposed that there are four obvious biochemical factors that may affect the metabolism of fluoroacetate: (1) the rate of conversion of fluoroacetate to fluorocitrate; (2) the sensitivity of aconitase to fluorocitrate; (3) the citrate transport system in mitochondria, and; (4) the ability to detoxify fluoroacetate [42, 43]. A study compared two distant populations of possums, one having prior exposure to fluoroacetate vegetation and the other having no prior exposure. No differences were found in the defluorination rate of liver extracts between the two populations [42]. Despite a number of other studies attempting to address the biochemical mechanisms for tolerance and fluoroacetate detoxification, there is still inadequate information available.

The soil bacterium *Streptomyces cattleya* is able to produce both fluoroacetate and fluorothreonine but has pathways that possibly confer resistance to these compounds [50]. A fluoroacetyl-CoA-specific thioesterase (FlK) in *S. cattleya* selectively hydrolyzes fluoroacetyl-CoA over acetyl-CoA and exhibits a 10^6^-fold higher catalytic efficiency for fluoroacetyl-CoA compared to acetyl-CoA [51]. The FlK gene is located in the same cluster as the C-F bond-forming fluorinase (flA), raising the probability that FlK-catalyzed hydrolysis of fluoroacetyl-CoA plays a role in fluoroacetate resistance in *S. cattleya* by inhibiting the entrance of fluoroacetyl-CoA into the TCA cycle [52].

## Degradation of fluoroacetate

Studies to isolate, purify and characterise fluoroacetate-detoxifying enzymes from animals have generally been unsuccessful and contradictory in their findings. Nonetheless, it is generally appreciated from early studies that the vast majority of fluoroacetate is defluorinated within the liver by an enzyme termed fluoroacetate specific defluorinase [53, 54]. This enzyme has been purified from mouse liver cytosol but it is distinct from multiple cationic and anionic glutathione S-transferase isozymes [55]. However, there has been no definitive classification of the enzyme [56]. The enzyme appears to act via a glutathione-dependent mechanism [57]. The focus of the most recent studies has been to determine the relationship between fluoroacetate specific defluorinase and glutathione S-transferase family enzymes to gain a better understanding of the mechanism of fluoroacetate detoxification.

Mead and co-workers [58] characterized a glutathione-dependent dehalogenation pathway in the liver of possum utilizing fluoroacetate as substrate. In the urine of fluoroacetate-treated animals, they found S-carboxymethylcysteine which indicates defluorination was catalyzed by an enzyme of the glutathione S-transferase group.

## Microbial aerobic degradation

Contrary to the animal studies on fluoroacetate detoxification, microbial degradation of fluoroacetate has been extensively studied. Moreover, the mechanism for aerobic fluoroacetate degradation is well characterised and documented [59–64]. Microorganisms from the soil have been identified with ability to aerobically degrade fluoroacetate. The bacterial communities involved in fluoroacetate degradation vary significantly depending on the areas studied. In Western Australia, species of *Bacillus, Pseudomonas, Aspergillus, Penicillium* and *Streptomyces* were isolated from soil in a of temperate climate [64], while *Burkholderiaceae, Ancylobacter* sp., *Paenibacillus *sp., *Staphylococcus *sp. and *Stenotrophomonas *sp. were isolated from the soil of Brazilian areas where the fluoroacetate-containing plants *Mascagnia rigida* and *Palicourea aenofusca* are found [65].

Microorganisms have also been isolated from bait containing the 1080 poison (fluoroacetate) that is used for vertebrate pest control [66]. Bacteria, particularly *Pseudomonas fluorescens*, were isolated from the 1080 bait when mixed with ground kangaroo meat, while both bacteria and soil fungi such as *Fusorium oxysporum* have been isolated from the bait mixed with oats [66, 67]. The bacteria and soil fungi degraded fluoroacetate in the presence and absence of another carbon source. However in the presence of peptone, degradation was higher.

In Western Australia, several microorganisms were isolated from soil with and without previous exposure to fluoroacetate. These included (*Aspergillus fumigatus*, *Fusarium oxysporum, Pseudomonas acidovorans, Pseudomonas fluorescens 1,* an unidentified *Pseudomonas* sp., *Penicillium purpurescens* and *Penicillium restriction.* These microbes can degrade fluoroacetate, presumably utilising it as a carbon source when grown in solution (2 to 89%) [67]. Recently, two other fluoroacetate degrading-bacteria were isolated from the Brazilian caprine rumen which had the ability to degrade fluoroacetate under aerobic conditions [68]. The bacteria were closely related to *Pigmentiphaga kullae* and *Ancylobacter polymorphus*. Fluoroacetate was degraded to fluoride ions, but the end products containing the carbon atoms from fluoroacetate were not discussed. Moreover, these bacteria might potentially be facultative anaerobes, and it was speculated that degradation occurred through the aerobic process.

**Fig. 4:**
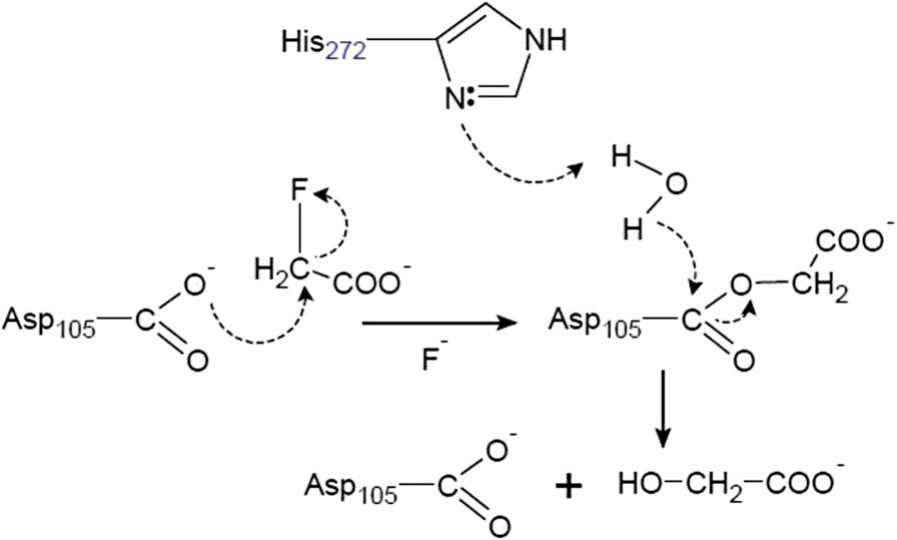
The mechanism of dehalogenation by fluoroacetate dehalogenase in *Delftia acidovorans*

Walker and Lien [59] were first to identify two fluoroacetate-degrading enzymes (initially termed haloacetate halidohydrolase) from *Pseudomonas* species and a fungus *Fusarium solani*. At the same time, a fluoroacetate dehalogenase was isolated from a fluoroacetate-dehalogenating bacterium in industrial wastewater, and tentatively named *Moraxella* sp. strain B [62]. It has now been reclassified as *Delftia acidovorans* strain B*.* Other soil bacteria which play a role in defluorination of fluoroacetate are *Burkholderia *sp. strain FA1, *P. fluorescens*, *Rhodopseudomonas palustris* CGA009 and different strains of *Pseudomonas* species [61, 66, 69, 70]. The fluoroacetate dehalogenase enzymes identified in some of these bacteria appear to degrade fluoroacetate via a similar mechanism, where an ester is produced as an intermediate which is hydrolyzed by a water molecule to form glycolate (Fig. 4).

In spite of their novel mechanisms, limited work has been conducted on these enzymes. The biochemical studies show (Table 1) relatively similar properties between these dehalogenases. All the bacterial enzymes have optimal activities at a slightly alkaline pH around pH 8.0 to 9.0 [59, 61, 69]. However, defluorinating activities in fungi have a wider optimal pH range, with pH 7-8 for *F. solani* compared to pH 5-8 for *F. oxysporium* [59, 67].

**Table 1:** Physical and biochemical properties of fluoroacetate dehalogenase isolated from different aerobic microorganisms

Microbial source	Number of genes ^a^	Gene location	Native enzyme sizes,kDa	Subunit Composition	Optimal pH	Optimal temperature	Reference
*Delftia acidovorans* strain B	2,*deH1, deH2*	Plasmid	67	Dimer	9.5	50	[60]
*Pseudomonas fluorescens* DSM 8341	N.D.	N.D.	32.5	monomer	8	30	[69]
*Burkholderia* sp. FA1	1,*fac-dex*	Chromosome	79	Dimer	9.5	N.D.	[61]
*Rhodopseudomonas palustris *CGA009	1,RPA1163 ^b^	Chromosome	N.D	Dimer	N.D.	N.D.	[70]
*Pseudomonas* sp. strain A	N.D.	N.D.	42	Monomer	9	50	[62]
*Pseudomonas* sp.	N.D.	N.D.	62	N.D.	8	N.D.	[59]
*Fusarium solani*	N.D	N.D.	62	N.D.	7-8	N.D.	[59]

^a^ gene names were described in parentheses

^b^ gene name identified in the form of locus tag

The thermal stability of these enzymes differs significantly depending on the species of the microorganisms. Fluoroacetate dehalogenase in *Pseudomonas sp.* from the New Zealand soil was shown to have higher thermal stability, approximately 55 °C, than the fluoroacetate dehalogenase in *F. solani* [59]. However, this notion of high thermal stability was not observed in some *Psuedomonas* species, *P. fluorescens* DSM 8341 was shown to have thermal stabilities to 30 °C [69].

The dehalogenases were shown to use water as the sole co-substrate, and no evidence indicates the involvement of metal ions in their catalytic activity [59, 71]. However, an increase in fluoroacetate degradation activity with addition of low concentration metals ion such as Mg^2+^, Fe^2+^ and Mn^2+^ has been demonstrated but higher concentration of these metals were inhibitory [69]. Although all the enzymes have a similar degradation mechanism, the size of these enzymes varies significantly. *Pseudomonas *sp. strain A and *P. fluorescens* enzymes are presumed to be monomers, and have an estimated molecular weight of 42 and 32.5 kDa, respectively. Conversely *Burkholderia *sp. FA1 and *D. acidovorans* strain B are dimers of two identical subunits with an estimated molecular mass of 79 and 67 kDa, respectively [61, 72].

All these enzymes release inorganic fluoride from fluoroacetate, but some also cleave chlorinated and brominated analogues, albeit at slower rates [59, 61, 73]. To date, *D. acidovorans* strain B is the only fluoroacetate-dehalogenating bacterium which harbours two haloacetate dehalogenase enzymes; Fluoroacetate dehalogenase H-1 (*dehH1*) and fluoroacetate dehalogenase H-2 (*dehH2*) which are encoded by two different genes on its 65 kb plasmid pUO1. Fluoroacetate dehalogenase H-1 acts predominately on fluoroacetate, while fluoroacetate dehalogenase H-2 has a broader range of substrate specificity for haloacetate, but not fluoroacetate [73].

Two other fluoroacetate dehalogenase enzymes which were purified and tested for their substrate specificities are fluoroacetate dehalogenases from *Burkholderia *sp. FA1 (Fac-dex) and *R. palustris* CGA009 (RPA1163) [61, 70]. When compared to DelH1 of *D. acidovorans* strain B, the two fluoroacetate dehalogenases were more specific to fluoroacetate than to other halogenated analogues [61, 70].

To date, the mechanism of fluoroacetate degradation by fluoroacetate dehalogenase has been extensively studied in *Burkholderia *sp. strain FA1 and *D. acidovorans* strain B [63, 70, 72, 74–76]. Several catalytic regions were identified by comparing the amino acid sequence with that of a haloalkane dehalogenase from *Xanthobacter autotrophicus* [60], and the specific amino acids have been identified by mutagenic studies [63]. It has been found that the active site of the H-1 enzyme contains a conserved Asp105 and His272.

In the initial steps of the pathway for fluoroacetate degradation to glycolate, the carboxylate group of Asp105 acts as a nucleophile to form an ester intermediate around the beta carbon atom of fluoroacetate to displace the fluorine atom [63, 75]. Then the acetate intermediate is hydrolysed by a deprotonated water molecule formed by a conserved His272. The net result of the reaction is a displacement of a fluoride ion producing glycolate and regeneration of the carboxylate group belonging to Asp105 (Fig. 4).

The catalytic sites of *D. acidovorans* strain B are also conserved as Asp105 and His271 in *Burkholderia *sp. strain FA1 [72]. Moreover, release of fluoride was found to be stabilised by the hydrogen bonds to His149, Trp150 and Tyr212 of *Burkholderia *sp. strain FA1 [75]. This stabilisation effect reduces the activation barrier, where the energy required to cleave the C-F bond was calculated to be only 2.7 kcal/mol, despite the strong C-F bond. A similar structure was also noted in the fluoroacetate dehalogenase from *R. palustris* CGA009 [70].

Due to the fact that the fluoroacetate dehalogenase of *Burkholderia *sp. strain FA1 has a preference for fluoroacetate compared to chloroacetate, the substrate specificity was tested using this enzyme [76]. Using docking stimulations and quantum mechanics/molecular mechanics (QM/MM), Nakayama and colleagues [76] managed to show that fluoroacetate and chloroacetate were incorporated into the active site of fluoroacetate dehalogenase in different conformations. Moreover, the hydrogen bonds of the chloroacetate-enzyme complex do not sufficiently reduce the activation barrier for chloroacetate, which is in a good agreement with the observed high specificity of this enzyme towards fluoroacetate.

Li et al. [77] worked on the structural requirements for defluorination by fluoroacetate degalogenase or FAcD (from bacterium *Rhodopseudomonas palustris* CGA009, PDB code 3R3V) in enabling defluorination rather than dechlorination. They have shown that conformational variations relating to neutrally charged histidine are Hsd155 and Hse155 may cause differences in enzymatic preference. They found that the structure FAcDHse155 is more energetically feasible than the structure FAcDHsd155 for enzyme FAcD, whereas FAcDHse155 prefers defluorination rather than the dechlorination process. Besides the residues Arg111, Arg114, His155, Trp156, and Tyr219, the important role of residues His109, Asp134, Lys181, and His280 during the defluorination process were also emphasized in their experiment. In addition, they found that conformational variations may cause different enzymatic preferences toward competitive pathways.

## Microbial anaerobic degradation

Compared with aerobic degradation of fluoroacetate, there is a lack of studies on the isolation of anaerobic microorganisms that have the ability to degrade fluoroacetate. However recently, a native bacterium from the Australian bovine rumen was isolated using anaerobic agar plates containing fluoroacetate as a carbon source [1]. This bacterium, strain MFA1, which belongs to the Synergistetes phylum has the ability to degrade fluoroacetate, producing fluoride and acetate, as opposed to glycolate from aerobic fluoroacetate-degrading bacteria. Similar observations were noted from other studies on anaerobic degradation of trifluoroacetic acid in anoxic sediments, where acetate was produced from the degradation of this compound [78, 79]. Moreover, similar mechanisms were also noted with anaerobic dechlorinating bacteria. An anaerobic microbial enrichment culture containing *Dehalococcoides ethenogenes* 195 was capable of completely dechlorinating tetrachloroethene to chlorides and ethene [80].

Acetate is not used by strain MFA1 for growth, unlike aerobic fluoroacetate dehalogenating bacteria which utilise the end product, glycolate, as an energy source. Strain MFA1 appears to degrade fluoroacetate via the reductive dehalogenation pathway utilising it as terminal electron acceptor rather than a carbon source. Reductive dehalogenation occurs in anaerobic bacteria, where a halogen substituent is released from a molecule with concurrent addition of electrons to that molecule [81].

There appeared to be a consumption of hydrogen and formate during the growth of strain MFA1 in fluoroacetate [1]. This observation was also noted from reductive dehalogenation of other halogenated compounds in anoxic environment. A net loss of hydrogen was measured from anoxic sediment microcosms dosed with various halogenated compounds [82], and hydrogen was consumed by a *Dehalococcoides ethenogenes* strain 195 with degradation of tetrachloroethene and vinyl chlorides to ethene [83]. However, there is not yet any enzyme identified in strain MFA1 responsible for the degradation of fluoroacetate.

## Biotechnological-derived methods for fluoroacetate detoxification in cattle

There have been several attempts to reduce the toxic effects of fluoroacetate in ruminant livestock production. A biotechnological approach to the problem did provide some evidence that detoxifying fluoroacetate by microbial metabolism was possible in the rumen [84]. Gregg and colleagues [84] transformed the rumen bacterium *Butyrivibrio fibrisolvens* with the fluoroacetate dehalogenase gene (DelH1) from *Delfitia acidovorans* strain B, and the recombinant bacteria demonstrated active dehalogenation of fluoroacetate in vitro.

The fluoroacetate dehalogenase H1 gene from *D. acidovorans* strain B was incorporated into the plasmid pBHf for transfection into *Butyrivibrio fibrisolvens* [84]. The transfection was relatively stable, with the pBHf plasmid remaining detectable after 500 generations under non-selective conditions. Gregg and colleagues [84] also performed an in vitro study, where a growing population of the recombinant bacterium was able to release fluorine from fluoroacetate at the rate of 9.9 nmol/min/mg [84]. However, dehalogenase activity was not detected outside the bacterial cells, and so it was predicted that fluoroacetate in the media diffused readily into the cells [84]. The genetically modified *B. fibrisolvens* strain expressed dehalogenase enough to detoxify fluoroacetate from the surrounding medium at a rate of 10 nmol/(min·mg) bacterial protein in in vitro testing. The plasmid that carries the dehalogenase gene appears to be very stable and was retained by 100% of the transformed bacteria after 500 generations of growth in non-selective media [84].

In an in vivo study conducted by Gregg and colleagues [85], one group of sheep were inoculated with the recombinant bacteria before being fed fluoroacetate-injected snow-peas, while a control group was not inoculated with the recombinant bacteria. This study showed a significant difference between groups, where the inoculated sheep appeared to be relatively normal despite a 0.4 mg dose of fluoroacetate per kg of animal, while the control sheep died of the fluoroacetate poisoning [85]. The modified bacteria were able to colonise the rumens of two sheep and were shown to persist for an experimental period of 5 months.

In another in vivo study conducted using 20 Angus steers, animals orally inoculated with seven different strains of *Butyrivibrio fibrisolvens* (*B. fibrisolvens* 0/10, 10/1, 85, 149/83, 156, 291, 52/10 strains respectively) containing the plasmid (pBHf)-bearing the fluoroacetate dehalogenase gene DelH1 did not develop the acute symptoms of fluoroacetate toxicity compared to the controls [86]. PCR analysis of rumen fluid collected at 7, 12 and 15 days post-inoculation confirmed the presence of the recombinant bacteria in the rumen at 10^4^ to 10^7^ cells/ mL. Post-mortem PCR analysis of the rumen fluid from all test animals showed approximately 10^6^ colony forming units (CFU) per mL of recombinant *B. fibrisolvens* for several of the strains*,* 20 days after inoculation [86]. The dose of recombinant bacteria used was able to significantly diminish the effects of fluoroacetate poisoning. Therefore, these in vivo tests showed significant protection of livestock from fluoroacetate using the recombinant bacteria approach. However, in Australia, this technology has not been adopted because approval has not been granted due to strict government regulations regarding release of genetically modified organisms.

In order to prevent animals from unintentional fluoroacetate poisoning, one of the therapies involves the adsorption of fluoroacetate with activated charcoal or other resins. These agents were investigated for their abilities to absorb fluoroacetate from gastrointestinal fluid, thus potentially preventing the conversion of fluoroacetate to fluorocitrate [87]. Moreover, the doses of 2 g/kg of such resins are impractical for preventing fluoroacetate poisoning in livestock. Acetate donor therapy has also been investigated as a treatment for poisoning. Early studies on the effect of fluoroacetate poisoning revealed that fluoroacetate inhibits acetate metabolism in poisoned animals [88]. This led to other studies to investigate whether acetate in the animal at high concentration would provide protection to the animals from fluoroacetate poisoning [89]. This treatment was only effective when provided immediately after the ingestion of the toxin and therefore not practical for treating grazing livestock due to limited surveillance of animals in a rangeland production system. In some cases, animals have died after consumption of fluoroacetate due to the severity of symptoms caused by the depletion of tissue citrate. Therefore, by relieving the symptoms of fluoroacetate poisoning using citrate therapy, researchers have been able to enhance the survival rate of poisoned animals [90]. However, these symptom-reversing therapies would need to be administrated immediately to the poisoned animals to show any effect. Furthermore, some of the poisoned animals in these studies died of other complications even though the major symptoms were suppressed [90].

## Rumen microbial transfer

*Amorimia pubiflora* is one of the main causes of fluoroacetate poisoning in Brazil. In a recent study researchers were able to induce resistance to toxicity by feeding non-toxic doses of this plant to sheep. In addition transferring rumen contents from the resistant animals to naïve sheep was able to confer protection from toxicity [91].

## Conclusions

To date, attempts to prevent fluoroacetate toxicity have been unsuccessful except for physically preventing access to toxic plants in the grazing environment. Animal house studies have demonstrated in principle that rumen bacteria engineered to hydrolyse the toxin could prevent toxicity but approvals for the release of these organisms into the environment are unlikely due to current government regulatory restrictions. However the recent discovery of a naturally occurring rumen bacterium (Synergistetes strain MFA1) capable of degrading fluoroacetate may provide a biotechnological solution to the problem of toxicity in rangeland animals. Even though Synergistetes strain MFA1 appears to be ubiquitous throughout the digestive systems of animals such as emus, kangaroos and other cattle, they are present in low numbers which may limit their ability to protect the animal from a lethal dose of the toxin [1]. However it is possible that there are other rumen bacteria able to degrade fluoroacetate which are at higher abundance or could act in concert with other rumen microorganisms to ameliorate the full impact of the toxin. Therefore, further surveys for the presence of other fluoroacetate degrading rumen bacteria and studies on increasing the numbers of these bacteria and expression of the genes responsible for degrading the toxin seems a logical approach for developing a practical strategy to protect livestock from fluoroacetate poisoning. Recent studies demonstrating tolerance to toxicity by adapting the rumen microbiota to non-toxic doses of fluoroacetate further supports a ‘rumen detoxification’ approach.

## Abbreviations

AUD: Australian Dollar
CNS: Central nervous system
MM: Molecular mechanics
QM: Quantum mechanics
TCA: Tricarboxylic acid
